# Effect of a Er, Cr:YSGG laser and a Er:YAG laser treatment on oral biofilm-contaminated titanium

**DOI:** 10.1590/1678-7757-2020-0528

**Published:** 2020-11-30

**Authors:** So-Hyun Park, Ok-Joon Kim, Hyun-Ju Chung, Ok-Su Kim

**Affiliations:** 1 Chonnam National University School of Dentistry Dental Science Research Institute Gwangju Republic of Korea Chonnam National University, School of Dentistry, Dental Science Research Institute, Department of Periodontology, Gwangju, Republic of Korea.; 2 National University School of Dentistry Chonnam Department of Oral Pathology Gwangju Republic of Korea Department of Oral Pathology, National University, School of Dentistry Chonnam, Gwangju, Republic of Korea.; 3 Chonnam National University Hard-tissue Biointerface Research Center Department of Periodontology Gwangju Republic of Korea Chonnam National University, School of Dentistry, Hard-tissue Biointerface Research Center, Department of Periodontology, Gwangju, Republic of Korea.

**Keywords:** Biofilms, Dental implants, Laser therapy, Microbial viability

## Abstract

Implant surface decontamination is a challenging procedure for therapy of peri-implant disease.

**Objective:**

This study aimed to compare the effectiveness of decontamination on oral biofilm-contaminated titanium surfaces in Er:YAG laser, Er, Cr:YSGG laser, and plastic curette.

**Methodology:**

For oral biofilms formation, six participants wore an acrylic splint with eight titanium discs in the maxillary arch for 72 hours. A total of 48 contaminated discs were distributed among four groups: untreated control; decontamination with plastic curettes; Er, Cr:YSGG laser; and Er:YAG laser irradiation. Complete plaque removal was estimated using naked-eye and the time taken was recorded; the residual plaque area was measured and the morphological alteration of the specimen surface was observed by scanning electron microscopy. The total bacterial load and the viability of adherent bacteria were quantified by live or dead cell labeling with fluorescence microscopy.

**Results:**

The mean treatment time significantly decreased based on the treatment used in the following order: Er:YAG, Er, Cr:YSGG laser, and plastic curettes (234.9±25.4 sec, 156.1±12.7 sec, and 126.4±18.6 sec, P=0.000). The mean RPA in the Er, Cr:YSGG laser group (7.0±2.5%) was lower than Er:YAG and plastic curettes groups (10.3±2.4%, 12.3±3.6%, p=0.023). The viable bacteria on the titanium surface after Er, Cr:YSGG laser irradiation was significantly lower compared to the decontamination with plastic curette (P=0.05) but it was not significantly different from the Er:YAG laser irradiation.

**Conclusion:**

We found that Er:YAG laser and Er, Cr:YSGG laser irradiation were effective methods for decontaminations without surface alterations.

## Introduction

Current implant dentistry focuses on the maintenance of long-term stability of peri-implant tissue. As the popularity of dental implant therapy has increased, so have the reported cases of peri-implant diseases.^1^ Peri-implant mucositis is a reversible inflammatory reaction of soft tissues around functioning implants, whereas peri-implantitis is a non-reversible inflammatory process related to the loss of the supporting bone around functioning implants.^2^ According to a review of Lee, et al.^3^ (2017) peri-implant mucositis occurs in 46.83% of patients receiving dental implant therapy and in 29.48% of functioning implants, whereas peri-implantitis has been found in 19.83% of patients and in 9.25% of functioning implants.

As peri-implant infection is caused by biofilm formation on implant surfaces, it is mandatory to decontaminate the implant surfaces for treatment or maintenance of peri-implant tissue.^4^ Removal of all calcified deposits and plaque from the implant surface is challenging because of macroscopic structures and specific micro-surface topography that hampers the cleansing procedure.^5^ Several conventional instruments and approaches including plastic or titanium curettes, ultrasonic and air abrasive devices have been commonly used for removing biofilms from the dental implant surfaces.^6^ However, as these conventional approaches cannot remove bacteria stuck to the implant surfaces, adjunctive chemical irrigation agents have been clinically examined and they have been shown to improve post-treatment healing.^7^ Patianna, et al.^8^ (2018), in an *in vitro* study, showed that the use of 14% doxycycline gel efficaciously decontaminated both machined and sandblasted acid etched implants surface.

Apart from these conventional approaches, the choice of several lasers has been proposed to treat peri-implant diseases. Lasers with several wavelengths may have different clinical applications depending on the tissue affinity and the degree of penetration.^9^ According to the research of Mailoa, et al.^10^ (2014), some effective lasers for peri-implantitis treatment are still inadequate, the CO_2_ and Er:YAG lasers are the most studied lasers due to their high bactericidal ability, and lasers could be an adjunct in the treatment of peri-implantitis. Nd:YAG (Neodymium-doped Yttrium Aluminum Garnet) laser and CO_2_ lasers are not suitable, for they generate heat while treating peri-implant infection, altering or damaging the implant surface.^11^ Recently, diode laser, which are increasingly used in dentistry due to their excellent versatility and being cheaper than garnet laser, are employed as an adjunctive tool of non-surgical mechanical therapy or even used itself as a valuable tool for treating peri-mucositis and peri-implantitis,^12^^–^^16^ furthermore, they are used with certain precaution, maintaining a 3 mm distance from the surface, a continuous movement over the surface, a maximum power of 1W and preferably, a pulsed wave mode.^17^ Er:YAG (erbium-doped: yttrium, aluminum and garnet) lasers (wavelength of 2,940 nm) was proposed as a suitable irradiation for implant surfaces, since it does not significantly raise the temperature of implant body during irradiation.^18^ According to several experimental and clinical results, Er:YAG laser seems to remove bacterial deposit efficiently from both machined and rough-surface implants without damaging their surfaces,^19^^–^^22^ and they appear capable of restoring an adequate osteoconductivity of decontaminated surfaces.^23^ A pilot study by Leja, et al.^24^ (2013) determined the effect of irradiation with diode, carbon dioxide, and Er:YAG lasers on the surface temperature of bone-placed implant fixture, *in vitro* and their results presented a wide variability of temperature among lasers and settings. During laser irradiation, the critical threshold of 10°C can be reached after only 18 sec. Er,Cr:YSGG (erbium, chromium-doped: yttrium, scandium, gallium, garnet) lasers, at a wavelength of 2,780 nm, have also been reported to improve the decontamination of bacterial deposits from the implant.^25^ According to Schwarz, et al.^26^ (2006) at power setups of up to 2.5 W, Er. Cr:YSGG laser with a cone-shaped fiber tip supposedly cause no thermal damage to surfaces of the titanium implant. Furthermore, they reported that Er,Cr:YSGG laser significantly decreased the early oral biofilms growth on surfaces sandblasted with large grit alumina, and acid etched titanium surfaces in an energy- and time dependent-manner. Furthermore, Romanos, et al.^27^ (2006) in a *in vitro* study, showed that implant surface decontamination by an the Er,Cr:YSGG laser favors osteoblast attachment.

The purpose of the present study was to evaluate the effectiveness of Er:YAG laser and Er,Cr:YSGG laser on oral biofilm-contaminated titanium surfaces in comparison with plastic curettes.

## Methodology

### Subjects

Six periodontal and systemically healthy volunteers (one woman, five men; mean age, 28.8±2.2 years) were included in this study for formation of *in vivo* plaque biofilm. This study protocol was approved by the Institutional Review Board of Chonnam National University Dental Hospital (CNUDH-2014-008) and all participants provided an informed consent form. Inclusion criteria for participants were: (1) not using systemic antibiotics during the last 6 months, (2) good oral hygiene (O’Leary index <10%), (3) no signs of chronic periodontitis or any inflammatory conditions in the soft tissue, and (4) non-smokers.

### Instrumentation/Measurement

#### In vivo biofilm formation

**Figure 1:**
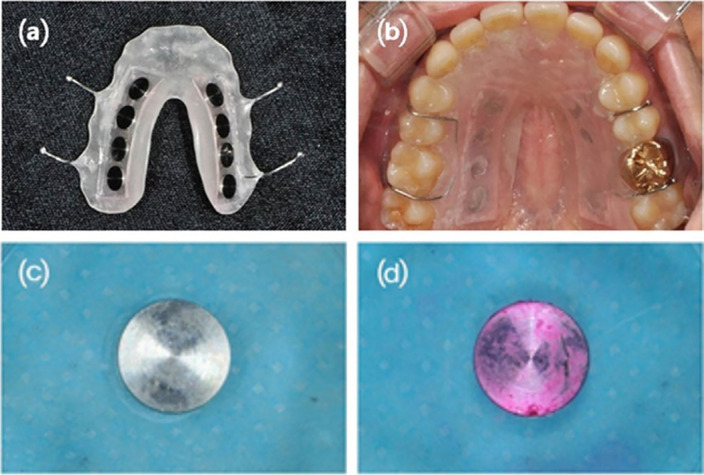
(a) fabricated splint. (b) Intraoral view of a splint with discs. Biofilms had been formed *in vivo* for 72 hours, sample (c) before and (d) after staining the biofilm with disclosing solution

Acrylic splints for the maxillary arch including eight titanium discs (Kobe Steel, Japan, commercially pure titanium grade II, diameter 6 mm thickness 2 mm), were fabricated for the collection of plaques (Figure 1a). The surface roughness (Ra) of titanium disc specimens was determined using a three-dimensional (3D) optical profiler (MV-E1000, NANO SYSTEM, Korea). Measurements were taken from an average of five different spots in each specimen (Ra=0.66 μm).

Acrylic splints with 8 mm x 30 mm rectangular hole of 3 mm depth on the left and right palatal side were manufactured and sterilized with ethylene oxide gas. After the titanium discs sterilization in a high pressure steam turbine, discs were attached in acrylic splints with flowable resin (G-aenial Flo, GC Co., Japan) with 1 mm distance to the palate, according to John, et al.^28^ (2014). By maintaining this distance, soft tissues and tongue influence could be excluded while ensuring a moist and nutritious environment.

Participants wore the splints for 72 hours, except when they manually brushed their teeth. Participants followed their regular diet during this period. Immediately after biofilm formation *in vivo*, the titanium discs were removed from the splint, water-washed, and dyed with disclosing solution (FD&C Red #28, Sultan Healthcare, USA). The disclosing solution was used to dye oral-biofilms growth in titanium surfaces (Figure 1d).

## Materials

**Figure 2:**
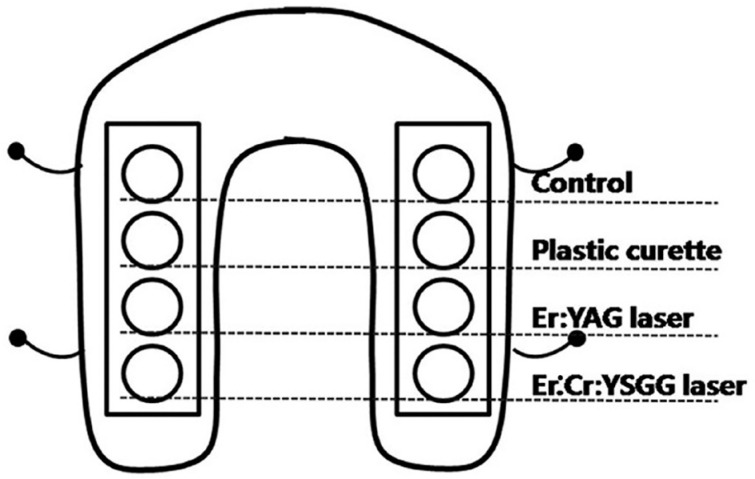
Diagram of experimental design on assigned treatment

Contaminated titanium discs were collected and each splint was divided into four sections with two titanium discs each, and they were assigned to the following groups (Figure 2): (1) untreated control (n=12); (2) decontamination with plastic curettes (n=12); (3) Er:YAG laser irradiation (n=12); and (4) Er,Cr:YSGG laser irradiation (n=12).

### Procedures

#### Treatment procedure

The following treatments were applied in all samples to completely remove the biofilm stained by disclosing the solution visible to the naked eye. In the plastic curette group, stained biofilms were removed using a plastic curette (Hu-Friedy Mfg. Co., USA) with saline irrigation. In the Er:YAG laser (wavelength of 2,940 nm, Anybeam E, BnB system, Korea) group, laser parameters were set at 50 mJ/pulse (8.92 J/cm^2^), 30 Hz, 150 μsec, water 30%, air 70%. Laser irradiation was performed in non-contact mode at a distance of 0.5 ~ 1 mm from the disc surface by a pain-ended cylindrical tip with a diameter of 850 μm to avoid any mechanical damage caused by contact mode according to Matsuyama, et al.^29^ (2003). In Er,Cr:YSGG laser (wavelength of 2,780 nm, Waterlaser MD, Biolase, USA) group, irradiation was performed at a setting of 2.5 W average power at 25 Hz, pulse duration 140 μsec (35.7J/ cm^2^), water 30%, air 30%.^26^ The laser beam was collimated by a cylindrical glass tip with 600μm diameter at 1 mm distance perpendicularly to the disc surface with non-contact mode. Irradiation was performed in a zigzag pattern. The operator was trained to perpendicularly irradiate the titanium surface via a cone-shaped fiber tip placed 1 mm above the surface. All treatments were conducted by a single trained operator. During cleansing treatment, the time taken to completely remove stained biofilms visible to the naked eye was also measured.

### Live/Dead bacteria labeling in combination with confocal fluorescence microscopy

To detach the residual biofilm from four specimens placed on the left side of each splint (total of 24 specimens), a vortex mixer (KMC-1300V, Vision scientific Co. Ltd, Korea) was used to immerse each specimen in 300 μL phosphate buffered saline (PBS, Mediatech, USA) for 20 seconds. The immersion time (20 seconds) was applied evenly to equalize the amount of biofilm being detached. The proportion of live or active bacteria (fluorescent green) and dead or inactive bacteria (fluorescent red) was determined by the Live/Dead BacLight bacterial viability kit (Molecular Probes, USA). The live/dead stain was prepared by diluting 0.5 μL of staining component A (SYTO 9) and 0.5 μL of staining component B (propidium iodine) in 300 μL of bacterial suspension. After thoroughly mixing and incubating in the dark for 15 minutes at room temperature, 200 μL of the suspension was carefully placed on a glass slide. Fluorescence emission was determined by fluorescence microscopy (Carl Zeiss MicroImaging GmbH, Germany). Images of three randomly selected sites for each specimen were captured with a digital camera connected to the microscope. The areas covered by live and dead bacteria were estimated as percentage of specific standard microscopic fields (1100 x 1100 μm=1.21 mm^2^) using Image J (National Institutes of Health, USA).

### Scanning electron microscopy (SEM)

Four discs located on the right side of each splint (24 specimens in total) were fixed in 2.5% glutaraldehyde for 2 hours at room temperature, for analyzing morphological changes of the surfaces and residual plaque areas (RPA). After fixing, specimens were washed three times with PBS (pH 7.4), and dehydrated with a series of graded ethanol solutions (30%, 60%, 95%, and 100%) for 15 minutes. Then, the specimens were dried in hexamethyldisilazane, sputter-coated with gold, and photographed using scanning electron microscopy (SEM: S-4700^®^, Hitachi, Japan). SEM was performed for each specimen at 30, 10,000 magnifications. RPA was measured as a percentage of biofilm by using Image J. The morphological changes of the specimen surfaces (e.g. melting, cracking, and crater formations) were observed by SEM imaging at 10,000 magnification.

### Statistical analyses

The data of this study are presented as mean ± standard deviation (SD). Statistical Package for the Social Sciences (SPSS ver.20.0, SPSS Inc., USA) was used for all analyses. Since non-normal distributions were assumed to occur, we analyzed data by applying non-parametric Kruskal-Wallis test for differences in treatment time, RPA, and the amount of dead and live bacteria adhered on surface in each treatment. Post hoc analysis with Mann-Whitney U test was performed using Bonferroni correction to test the differences between groups. *P*-values <0.05 were considered as statistically significant.

## Results

### Treatment time

**Figure 3:**
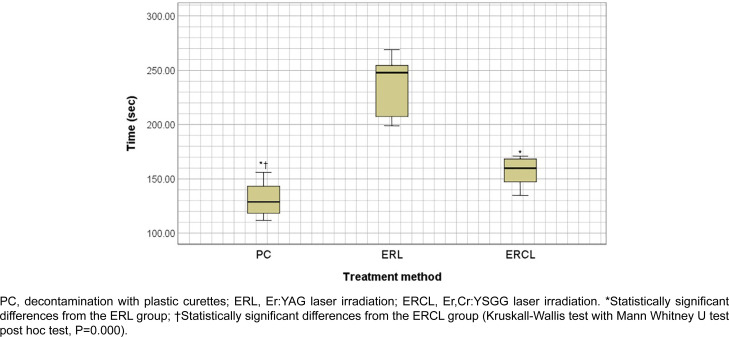
Mean treatment times (sec) of PC, ERL, and ERCL

The mean treatment times were statistically significant in each group (Figure 3). The mean treatment time was lower in Er:YAG laser group, followed by Er,Cr:YSGG laser, and plastic curette groups (234.9±25.4 sec, 156.1±12.7 sec and 126.4±18.6 sec, respectively; *P*=0.000 for all comparisons).

### Residual plaque areas

**Figure 4:**
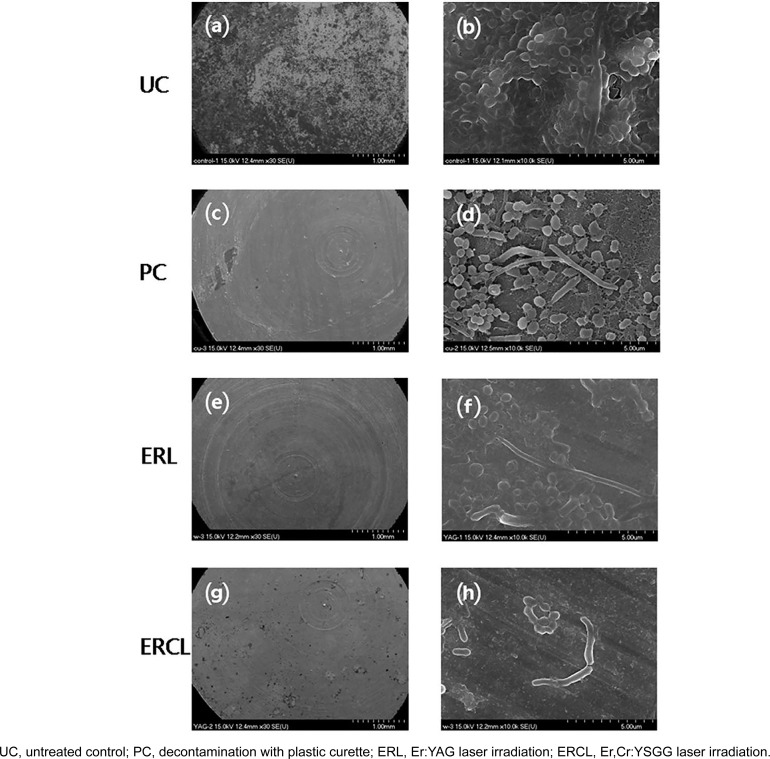
SEM images of surface (magnification ×30 ((a)(c)(e)(g)), ×10,000 ((b)(d)(f)(h)) after decontamination procedure on biofilms had been formed *in vivo* for 72 hours

**Figure 5:**
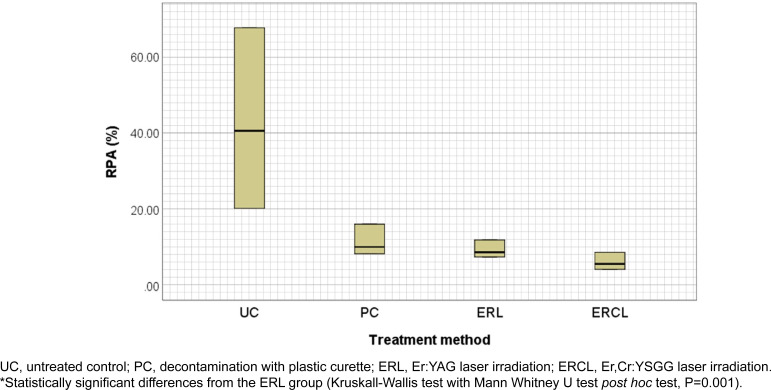
Mean residual plaque area (RPA) after decontamination procedure on biofilms had been formed *in vivo* for 72 hours

SEM images of each group show the surface morphology of treated titanium and bacteria from the remaining biofilm (Figure 4). In the naked eye observation, the biofilms stained by disclosing solution seemed to have been completely removed, however, SEM images indicated biofilm still remained. Following the treatments, typical laser-induced morphological alterations of titanium surface (e.g. melting, cracking, and crater formations) were not detected (10,000x magnification). After 72 hours, all samples were incompletely covered with oral biofilm. The mean early biofilm was at 49.8±18.7%. After treatment, the mean RPA of the Er,Cr:YSGG laser group (7.0±2.5%) was significantly lower than both plastic curette, and Er:YAG laser group (12.3±3.6%, 10.3±2.4%, respectively), *P*=0.001 for all comparisons (Figure 5).

### Proportion of live/dead bacteria

**Figure 6:**
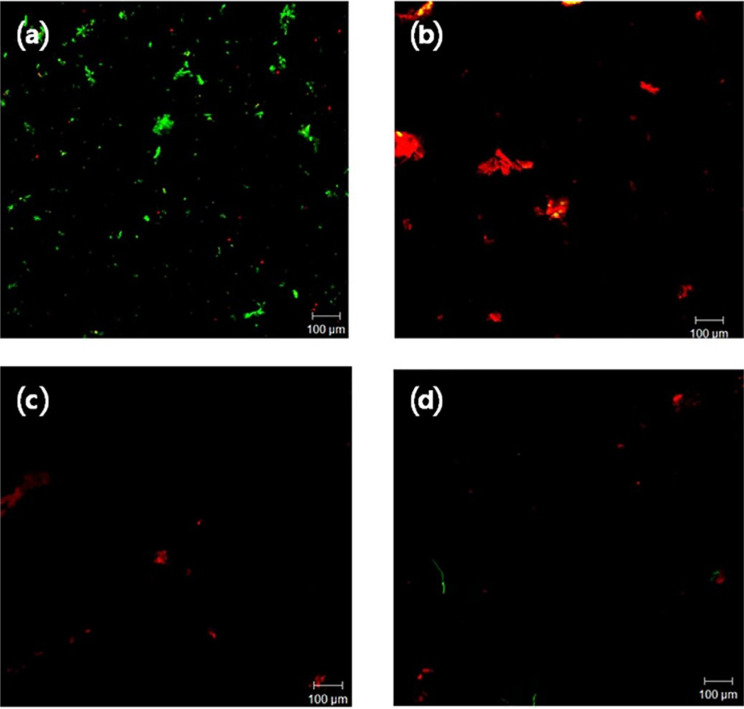
Micrograph of stained live/dead bacteria of the titanium surface after decontamination procedure on biofilms had been formed *in vivo* for 72 hours, observed with fluorescence microscope. Live bacteria (green) and dead bacteria (red) (scale bar=100 μm). (a) UC (untreated control), (b) PC (Decontamination with plastic curette), (c) ERL (Er:YAG laser irradiation), and (d) ERCL (Er,Cr:YSGG laser irradiation)

Fluorescence microscopy showed the proportions of live bacteria decreased and dead bacteria increased after laser treatment (Figure 6; Table 1), when compared to the control group. The total bacterial load on disc surfaces was significantly reduced after treatment in all groups (*P*=0.024 for all comparisons). Fewer total live and dead bacteria were covered in the two lasers than in plastic curette group (*P*=0.05 for both comparisons). The viable bacteria on the titanium surface after Er,Cr:YSGG laser irradiation was significantly lower compared to the decontamination with plastic curette use (p=0.05) and the difference was not significant after Er:YAG laser irradiation.

**Table 1:** Effectiveness of different decontamination procedure on biofilms had been formed *in vivo* for 72 hours indicated by live/dead fluorescence staining (mean±SD (%))

Treatment methods	Total bacteria	Dead bacteria	Vital bacteria	Ratio dead to total (%)
UC	61±15.7	6.9±2.6	54.1±13.8	11.2±3
PC	4.9±2.7	2.5±1.2	2.4±1.6	51.5±7.5
ERL	1.7±1.2*	1.3±0.8	0.4±0.5*	77.7±14.4
ERCL	1.5±0.8*	1.2±0.5	0.3±0.3*	83±13.6

UC, untreated control; PC, decontamination with plastic curette; ERL, Er:YAG laser irradiation; ERCL, Er,Cr:YSGG laser irradiation.

* Statistically significant differences from the PC group (Kruskal-Wallis test with Mann Whitney U test post hoc test, P<0.05).

## Discussion

This study examined the efficacy of Er,Cr:YSGG lasers compared to Er:YAG laser or plastic curette in the decontamination of titanium disc surfaces covered with oral biofilms. Using SEM and live/dead bacteria staining after decontamination on biofilms that had been formed *in vivo* for 72 hours we found that Er,Cr:YSGG laser was the most effective decontamination method.

These results show that homogenous plaque biofilms cover all titanium discs after 72 hours *in situ*. This agrees with previous studies investigating biofilms formation on different specimens, which showed a mature and homogenous biofilm of inserted disks after 24 hours.^30^^,^^31^ The splints used in this study were made similarly to that described in an aforementioned study.^30^ Surfaces of titanium discs in acrylic splints were positioned toward the palate at a distance of 1 mm for nutritious moist circumstances to provide favorable conditions for *in vivo* biofilm formation.

All decontamination methods used in this study decreased the residual plaque area. After cleansing, mean residual plaque area in the plastic curette group was higher than that in the Er:YAG laser and the Er,Cr:YSGG laser group. The results of previous investigations examining the effectiveness of different types of lasers as plaque removal methods are similar to ours: Er:YAG laser: 5.8±5.1%, Er,Cr:YSGG laser: 9.8±6.2%.^26^^,^^30^ However, in our study, the mean residual plaque area after decontamination with plastic curette was lower than previous study outcomes.^29^^,^^31^ According to the results from previous experiments, mean RPA after decontamination with a plastic curette ranged between 58.5±4.9% and 61.1±11.4%.^30^^,^^32^ This discrepancy is due to complete removal of bacterial plaque using disclosing agent without any time restrictions in our study. Furthermore, titanium surfaces without any structures (e.g. threads) enable easier access to a plastic curette.

The mean treatment time from the experiments in decreasing order: Er:YAG laser, Er,Cr:YSGG laser, and plastic curette group. The Er:YAG laser had the longest treatment time, concurring with previous study results.^30^ The treatment time for decontamination with an Er:YAG laser was longer than other methods 336±72s). The treatment time in this study was shorter compared to that study^30^ for the smaller sample size, with an area of 0.28 cm^2^ compared to 0.7 cm^2^. Furthermore, because of the smooth titanium surface, the area that can be removed at once with a plastic curette is larger. This explains why the plastic curette group presented the shortest treatment time. Treatment time in Er,Cr:YSGG laser was shorter than Er:YAG laser. One could explain that Er,Cr:YSGG laser was used at higher power settings in this study, and it has an ablative hydrokinetic process that enables more efficient decontamination and debridement.^25^

The quantification of adherent microorganisms based on biofluorescence are simple, precise, and reproducible. These systems are more convenient and reliable than the traditional methods of microbial quantification.^33^ The visual differences presented in this study between living and dead bacteria using live/dead staining techniques was significant in this investigation.^34^ SYTO9 and propidium iodide staining has shown to be better than other assays by providing an obvious difference between dead and active microorganisms without interfering with the background fluorescence.^35^ The use of fluorescence microscopy approaches enables the visualization of bacteria.^36^^,^^37^

In this study, anti-adherence activity of three treatments on biofilm contaminated titanium surface was examined by estimating the total bacterial load. The total bacteria on the titanium surface after two process of laser irradiation were significantly lower than after decontamination with plastic curette. This difference occurred because of the water source, which comes directly from the laser apparatus in irradiation, compared with passive irrigation in hand scaling. Additionally, the bactericidal effectiveness of laser irradiation was determined by estimating the percentage of dead to total bacteria after 72 hours of biofilm formation and decontamination procedure. All methods of treatment were capable of inactivating adhered microorganisms. We found that there was a higher ratio of dead to total bacteria in the laser group, which suggests that the laser had a bactericidal effect. The results of this experiment agree with previous study.^18^ According to the microbiological and microscopic result of previous study, Er:YAG laser is likely to have higher bactericidal possibility on implant surfaces.^18^ In our study, supragingival biofilm adhered on the titanium surfaces after a period of 72 hours was early non-mineralized plaque. Microbial composition might be different from subgingival plaque in the crevice of peri-implantitis site. Giannelli, et al.^23^ (2017) in their study used a bacterial plaque originated from the subgingival margin of diseased implants with sterile curettes and smeared on the surface of disks. Therefore, this issue about true pathogenic biofilm in peri-implantitis must be developed in further study.

Matsuyama, et al.^29^ (2003) suggested that for periodontal debridement, the radiant energy below 50mJ/pulses has usually been used, and, Er:YAG laser debridement of dental implant surfaces may be feasible without damaging their surfaces in the low irradiation setting. Furthermore, 30mJ/pulse, 30 Hz with water irrigation enabled effective removal of bacterial deposit and regions of calcification on the implant abutments without damaging their surfaces. Strever, et al.^38^ (2017) showed that an Er,Cr:YSGG laser effectively removes single-species biofilms on disks without cognizable physical injury, using clinically relevant power setting.

As long as we know, we are the first to report the effectiveness of Er. Cr:YSGG laser in compared to Er:YAG laser in removing *in vivo* biofilms on titanium surface by comparing quantitative or qualitative values obtained *in vitro* with conventional cleansing methods.

A limitation of this study is that biofilms were not formed in real rough surface of an implant but on a smooth titanium surface. Investigation of Rimondini, et al.^31^ (1997) showed that the surface roughness of specimens is the key factor in early *in vivo* plaque accumulation. According to Quirynen, et al.^39^ (1993) the surface roughness acts as a threshold in bacterial colony formation, preventing large bacteria from adhering to the surface with roughness lower than Ra=0.2 μm. We also found that the value of surface roughness (Ra=0.66 μm) of titanium disk surface was higher than the threshold even though it was machined surface, it did not prevent the bacterial colonization. In clinical situations, defect morphology around implants or poor restorations hamper the access to the region of interest. In fact, plastic curette is suitable for cleansing the platform site and upper structures of the implant, but if the tip width is larger than the distance between screw pitches of the implant, it is difficult to remove the biofilm, and may be left fine plastic remnants when used on rough-surface implants. Therefore, further research is necessary to clarify bacterial adhesion and method of decontamination on rough surface implant surfaces in clinically simulated situations. Considering that the laser energy at target may differ from the set in the control panel, especially for laser devices using optic fibers, direct measurement of beam energy of both Er:YAG and Er. Cr:YSGG lasers are necessary to perform by a power meter. It is important to provide reliable information on the delivered energy to achieve optimal decontamination without inducing unwanted alterations of implant surface. Also, an infrared thermal camera could also be helpful to monitor temperature at the targeted surface during laser irradiation.

## Conclusion

Considering the limit of this *in vitro* study, the effectiveness of Er:YAG laser and Er,Cr:YSGG laser in cleansing the contaminated biofilm on titanium surface was evaluated comparing it to the plastic curette in treatment time, SEM image, and live/dead bacteria staining. Considering that Er:YAG lasers and Er,Cr:YSGG laser did not alter the surface, they were more effective methods of decontamination on contaminated titanium surface than the plastic curette.

## Abbreviations

Er,Cr:YSGG: erbium, chromium-doped: yttrium, scandium, gallium, garnet
Er:YAG: erbium-doped: yttrium, aluminum, and garnet
RPA: residual plaque area
Nd:YAG: neodymium-doped yttrium aluminum garnet
PBS: phosphate buffered saline
SEM: scanning electron microscopy
